# Platelet number and interleukin-6 correlate with VEGF but not with bFGF serum levels of advanced cancer patients

**DOI:** 10.1038/sj.bjc.6690437

**Published:** 1999-05

**Authors:** R Salgado, P B Vermeulen, I Benoy, R Weytjens, P Huget, E Van Marck, L Y Dirix

**Affiliations:** Angiogenesis Group, Oncological Centre, Oosterveldlaan 24, Wilrijk-Antwerp, B2610, Belgium; Department of Pathology, University of Antwerp (UIA), Wilrijk, B2610, Belgium

**Keywords:** angiogenesis, interleukin-6, blood platelets, VEGF

## Abstract

We have compared the platelet number and the serum concentration of vascular endothelial growth factor (VEGF), basic fibroblast growth factor (bFGF) and interleukin-6 (IL-6) in 80 blood samples of 50 patients with advanced cancer. We have also measured the mitogenic effect of patient sera on endothelial cells in vitro in order to estimate the biological activity of serum VEGF. Serum VEGF concentration correlated with platelet number (*r* = 0.61; *P* < 10^−4^). Serum IL-6 levels correlated with platelet count (*r* = 0.36; *P* < 10^−3^), with serum VEGF levels (*r* = 0.55; *P* < 10^−4^) and with the calculated load of VEGF per platelet (*r* = 0.4; *P* = 3 × 10^−4^). Patients with thrombocytosis had a median VEGF serum concentration which was 3.2 times higher (*P* < 10^−4^) and a median IL-6 serum level which was 5.8 times higher (*P* = 0.03) than in other patients. Serum bFGF did not show an association with any of the other parameters. Patient sera with high VEGF and bFGF content stimulated endothelial cell proliferation significantly more than other sera (*P* = 4 × 10^−3^). These results support the role of platelets in the storage of biologically active VEGF. Platelets seem to prevent circulating VEGF from inducing the development of new blood vessels except at sites where coagulation takes place. IL-6, besides its thrombopoietic effect, also seems to affect the amount of VEGF stored in the platelets. This is in accordance with the indirect angiogenic action of IL-6 reported previously. The interaction of IL-6 with the angiogenic pathways in cancer might explain the stimulation of tumour growth occasionally observed during IL-6 administration. It also conforms to the worse outcome associated with high IL-6 levels and with thrombocytosis in several tumour types and benign angiogenic diseases. © 1999 Cancer Research Campaign


*British Journal of Cancer* (1999) **80**(5/6), 892–897
© 1999 Cancer Research Campaign
Article no. bjoc.1998.0437

### Platelet number and interleukin-6 correlate with VEGF but not with bFGF serum levels of advanced cancer patients

**R Salgado[1], PB Vermeulen[1,2], I Benoy[1], R Weytjens[1], P Huget[1], E Van Marck[1,2] and LY Dirix[1]**

[1]Angiogenesis Group, Oncological Centre, A.Z. St.-Camillus/St.-Augustinus, Oosterveldlaan 24, B2610 Wilrijk-Antwerp, Belgium; [2]Department of Pathology, University of Antwerp (UIA), B2610 Wilrijk, Belgium

**Summary** We have compared the platelet number and the serum concentration of vascular endothelial growth factor (VEGF), basic fibroblast growth factor (bFGF) and interleukin-6 (IL-6) in 80 blood samples of 50 patients with advanced cancer. We have also measured the mitogenic effect of patient sera on endothelial cells in vitro in order to estimate the biological activity of serum VEGF. Serum VEGF concentration correlated with platelet number ($r = 0.61$; $P < 10^{-4}$). Serum IL-6 levels correlated with platelet count ($r = 0.36$; $P < 10^{-3}$), with serum VEGF levels ($r = 0.55$; $P < 10^{-4}$) and with the calculated load of VEGF per platelet ($r = 0.4$; $P = 3 \times 10^{-4}$). Patients with thrombocytosis had a median VEGF serum concentration which was 3.2 times higher ($P < 10^{-4}$) and a median IL-6 serum level which was 5.8 times higher ($P = 0.03$) than in other patients. Serum bFGF did not show an association with any of the other parameters. Patient sera with high VEGF and bFGF content stimulated endothelial cell proliferation significantly more than other sera ($P = 4 \times 10^{-3}$). These results support the role of platelets in the storage of biologically active VEGF. Platelets seem to prevent circulating VEGF from inducing the development of new blood vessels except at sites where coagulation takes place. IL-6, besides its thrombopoietic effect, also seems to affect the amount of VEGF stored in the platelets. This is in accordance with the indirect angiogenic action of IL-6 reported previously. The interaction of IL-6 with the angiogenic pathways in cancer might explain the stimulation of tumour growth occasionally observed during IL-6 administration. It also conforms to the worse outcome associated with high IL-6 levels and with thrombocytosis in several tumour types and benign angiogenic diseases.

**Keywords:** angiogenesis; interleukin-6; blood platelets; VEGF

An interim conclusion of the ongoing translational research concerning tumour angiogenesis is that obtaining quantitative information of this process in cancer patients seems to add prognostic and predictive power to the currently used tumour staging systems (Gasparini, 1996). Angiogenesis is an element of the stromal reaction to tumour cell populations and supports their growth (Folkman, 1990), invasion (Skobe et al, 1997) and metastasis (Liotta and Saidel, 1974). Although counting individual microvessels on immunostained tissue sections is feasible and informative (Vermeulen et al, 1996), a more observer-independent and dynamic method of quantifying angiogenesis in cancer patients is based on the assessment of circulating angiogenic factor concentrations.

Serum levels of basic fibroblast growth factor (bFGF) predicted accelerated growth of lung metastases in patients with renal cell carcinoma (Duensig et al, 1995). In advanced colorectal cancer, elevated serum bFGF and serum vascular endothelial growth factor (VEGF) levels were closely associated with short estimated volume doubling times of the metastases (Dirix et al, 1996). Serum angiogenic factor levels might also be used to select patients for a specific treatment. In metastatic renal cell carcinoma patients, elevated serum levels of bFGF predicted response to interferon alpha (IFN-α) treatment (Vermeulen et al, 1997). In colorectal cancer, preoperative serum VEGF measurements correlated with T stage and the extent of nodal involvement (Kumar et al, 1998). Evaluation of response to treatment might be another application of assessing circulating angiogenic factors in cancer patients. Patients with response to treatment had less frequently elevated bFGF and/or VEGF serum levels than patients with progressive tumours of various histological types (Dirix et al, 1997). Survival of cancer patients has been estimated using pretreatment serum angiogenic factor measurements. High VEGF concentrations were associated with shorter survival in non-Hodgkin's lymphoma (Salven et al, 1997) and in small-cell lung cancer (Salven et al, 1998). Elevated serum bFGF predicted a worse outcome in renal cell carcinoma patients (Dosquet et al, 1997).

Recently, preliminary evidence for VEGF storage by platelets in cancer patients has been described (Verheul et al, 1997; Banks et al, 1998). Serum VEGF concentrations correlated with platelet numbers in breast cancer patients during chemotherapy. After induction of platelet aggregation, the VEGF content in the supernatant of the platelet suspension was significantly increased (Verheul et al, 1997). Comparable results have been reported by Banks et al (1998). Activated platelets release VEGF together with β-thromboglobulin, suggesting that VEGF resides in the α-granules of platelets (Wartiovaara et al, 1998). Although megakaryocytes in the bone marrow produce VEGF (Möhle et al, 1997), the presence of VEGF mRNA and VEGF protein has been demonstrated to be up-regulated in several tumour types (Brekken

*Received 28 August 1998*
*Revised 30 November 1998*
*Accepted 9 December 1998*

*Correspondence to:* LY Dirix, Angiogenesis Group – Experimental Oncology, Oncological Centre

**Table 1** Correlation coefficient *r* and *P*-value of the correlation analysis of the different sets of variables

| | VEGF *r*/*P* | IL-6 *r*/*P* | Platelet *n* *r*/*P* |
|---|---|---|---|
| bFGF | 0.06/0.6 | 0.03/0.8 | 0.12/0.3 |
| VEGF | – | **0.55/<$10^{-4}$** | **0.61/<$10^{-4}$** |
| IL-6 | – | – | **0.36/$10^{-3}$** |

Platelet n = platelet number. Significant correlations are printed in bold.

et al, 1998). Therefore, an important source of circulating VEGF in cancer patients is presumably the tumour tissue. The hypothesis is that fast-growing tumours, besides producing angiogenic factors such as VEGF, also release thrombopoietic cytokines. An increase in the number of circulating platelets could then, by the uptake of free VEGF, focus the effect of this angiogenic factor to sites where coagulation takes place, e.g. a wound or a tumour.

Both thrombopoietin (Kaushansky, 1995) and interleukin-6 (IL-6) (Ishibashi et al, 1989) potently stimulate platelet production. Recombinant human thrombopoietin and IL-6 proteins have been used in clinical trials in cancer patients (Weber et al, 1993; Vadhan-Raj et al, 1997). Transient stimulations of tumour growth have been observed during IL-6 treatment, which disappeared immediately upon cessation of IL-6 treatment (Ravoet et al, 1994). IL-6 is produced in malignant tumours and in inflammatory tissues (Rak et al, 1996). IL-6 is up-regulated by hypoxia (Yan et al, 1995), like VEGF and other angiogenic factors. IL-6 expression is elevated in healing wounds (Mateo et al, 1994). Response elements for IL-6 signalling are present upstream to the transcription initiation site of VEGF (Cohen et al, 1996). Thrombocytosis is a clinical sign of malignant mesothelioma progression and has been associated with high levels of circulating IL-6 (Nakano et al, 1998). In inflammatory bowel disease, independent studies have shown that exacerbation of disease coincided with high serum levels of angiogenic factors (Bousvaros et al, 1997), with high serum levels of IL-6 (Bross et al, 1996) and with high platelet numbers (Lake et al, 1978).

Given these arguments, we compared the serum VEGF, bFGF and IL-6 concentrations with the platelet numbers in 80 samples from 50 patients with various types of metastatic cancer. To estimate the biological meaning of serum angiogenic factor levels we also assessed whether high serum VEGF and bFGF concentrations concurred with a mitogenic effect on human endothelial cells in vitro.

### PATIENTS AND METHODS

### Patients

Of advanced cancer patients, 10 ml of venous blood were drawn into a serum separator tube (type vacutainer code 607213, Becton-Dickinson) and immediately centrifuged at 3000 r.p.m for 10 min. Thereafter the serum was separated and aliquoted in 1.0-ml fractions and stored at –80°C. Eighty samples were randomly selected from this serum collection, related to 50 patients. Twenty-six patients had a metastatic breast adenocarcinoma, seven a metastatic renal carcinoma, six a metastatic ovarian adenocarcinoma, five a metastatic colorectal adenocarcinoma, two a high-grade lymphoma, one a high-grade testis tumour, one a metastatic small-cell lung tumour, one a malignant fibrous histiocytoma and one had a metastatic carcinoid tumour.

### Methods

Serum levels of bFGF and VEGF were determined using two enzyme-linked immunosorbent assay (ELISA) kits of R&D Systems (Minneapolis, MN, USA; Quantikine High Sensitivity human FGF basic and Quantikine human VEGF). Within-assay reproducibility has been tested before (Dirix et al, 1997). Due to exhaustion of the serum aliquots, bFGF could not be measured in four patients.

Serum levels of Il-6 were determined with an ELISA kit of R&D Systems (Quantikine human IL-6). The assay employs a quantitative sandwich enzyme immunoassay technique combining a bound monoclonal antibody specific for IL-6 with an unbound enzyme-linked polyclonal antibody specific for IL-6. Within-assay reproducibility was evaluated on duplicates. When 40 serum samples from apparently healthy individuals were evaluated by R&D Systems, 83% had an IL-6 content of less than 3.13 pg $ml^{-1}$ or the detection limit. Comparably, IL-6 was undetectable, or was at the limit of detection of the assay, i.e. 4 pg $ml^{-1}$, in the serum of normal subjects in the study of Nakano et al (1998). Thrombocytosis was defined as a platelet number above $400 \times 10^9$ $1^{-1}$. In four patients, platelet counts were not performed on a sample taken at the time of serum sampling and were therefore not used.

### Endothelial cell proliferation assay

Human umbilical vein endothelial cells (HUVEC) were isolated according to Jaffe et al (1973) and seeded onto gelatin-coated plastic. They were maintained in M199 medium supplemented

**Table 2** The comparison of bFGF, VEGF and IL-6 serum levels in patients without thrombocytosis versus patients with thrombocytosis and according to the median platelet number (platelet *n*)

| Platelet *n* ($10^9$ $l^{-1}$) | bFGF (pg $ml^{-1}$) | VEGF (pg $ml^{-1}$) | IL-6 (pg $ml^{-1}$) |
|---|---|---|---|
| ≤ 400 (*n* = 62) | 5.43 ± 6.68 (2.52) | 351 ± 297 (233) | 8.67 ± 22.35 (1.50) |
| > 400 (*n* = 14) | 9.02 ± 9.19 (6.72) | 822 ± 464 (739) | 28.54 ± 47.51 (8.72) |
| *P*-value | 0.10 | < $10^{-4}$ | 0.03 |
| ≤ median (*n* = 40) | 5.81 ± 7.12 (2.98) | 254 ± 186 (191) | 5.78 ± 7.13 (1.50) |
| > median (*n* = 36) | 6.46 ± 7.57 (4.06) | 642 ± 431 (562) | 19.60 ± 40.96 (3.96) |
| *P*-value | 0.83 | < $10^{-4}$ | 0.08 |

Mean concentrations ± standard deviation (median) are given. Mann–Whitney *U*-test.

© Cancer Research Campaign 1999 

**Table 3** The comparison of bFGF and VEGF serum levels according to platelet number (platelet *n*) and IL-6 serum levels

| | bFGF (pg ml$^{-1}$) | VEGF (pg ml$^{-1}$) |
|---|---|---|
| Platelet *n* low **and** IL-6 low (*n* = 23) | 5.31 ± 7.70 (1.39) | 161 ± 81 (180) |
| Platelet *n* high **or** IL-6 high (*n* = 30) | 5.73 ± 5.67 (4.54) | 384 ± 263 (304) |
| Platelet *n* high **and** IL-6 high (*n* = 23) | 7.38 ± 8.70 (3.09) | 785 ± 423 (760) |
| *P*-value | 0.52 | < 10$^{-4}$ |

For platelet number the cut-off value was the median. For IL-6 the cut-off value was the detection limit. Mean concentrations ± standard deviation (median) are given. Kruskal–Wallis test.

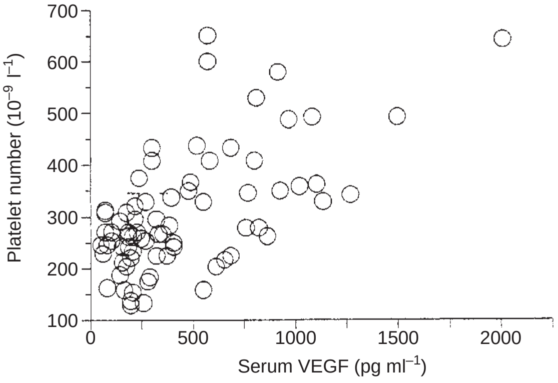

**Figure 1** Correlation between platelet number and serum VEGF concentration (*n* = 76; *r* = 0.61; *P* < 10$^{-4}$)

with 20% fetal calf serum (FCS), ECGS 0.01 mg ml$^{-1}$ (Sigma), heparin 1 U ml$^{-1}$ and antibiotics. First to second passage cells were used. All cultures were maintained in a humidified 5% carbon dioxide incubator at 37°C. Subcultures were obtained by enzyme treatment (0.05% trypsin, 0.2% EDTA).

For the proliferation assay, 20 000 cells were put into one well (24-well plate) and allowed to adhere and proliferate for 24 h. The medium was then changed. In the control wells the standard medium was put with 20% FCS. In the other wells, the FCS was replaced by patient serum (20%). After 24 h the endothelial cells were harvested and the viable cells were selected by trypan blue dye exclusion and counted in a haematocytometer. All experiments were performed in triplicate. The results were expressed as the ratio of the number of HUVEC in the wells with the patient serum versus the number of HUVEC in control wells (percentage). Twenty serum samples of cancer patients and five samples of healthy volunteers were randomly taken from the serum collection of our laboratory. Serum bFGF and VEGF were determined as described before.

### Statistical analysis

Statistical analysis was performed with the Statview 4.51 software application (Abacus Concepts) on an Apple Macintosh personal computer. The relation between continuous variables was analysed by a correlation analysis. Comparisons of continuous variables in different subgroups were performed by Mann–Whitney *U*-test and Kruskal–Wallis test. The differences in fractions of patients/samples were analysed by a $\chi^2$ test. A *P*-value < 0.05 was considered to be significant.

### RESULTS

Forty-two (53%) serum samples from 35 (70%) patients had a measurable IL-6 content, which was higher than the detection limit of 3.12 pg ml$^{-1}$. The mean coefficient of variation of the duplicate analysis of these samples was 5.7% (*n* = 42; range 0.53–29.50). When IL-6 was not detectable in a serum sample, the value of 1.5 pg ml$^{-1}$ was entered for further analysis, i.e. approximately 50% of the detection limit.

Mean bFGF level was 5.96 pg ml$^{-1}$ (standard deviation (s.d.) 7.20; median 3.07; range 0.11–33.0; *n* = 76). Mean VEGF level was 453 pg ml$^{-1}$ (s.d. 381; median 314; range 40–2000; *n* = 80). Mean IL-6 level was 13.74 pg ml$^{-1}$ (s.d. 30.42; median 3.42; range 1.50–170.40; *n* = 80). Mean platelet number was 307 10$^{9}$ l$^{-1}$ (s.d. 115; median 276; range 132–655; *n* = 76). Fourteen of 76 (18%) samples from 13 of 46 (28%) patients showed thrombocytosis.

Results of the correlation analysis of the different sets of variables are given in Table 1. The strongest correlations were present between serum VEGF and platelet number (*n* = 76; *r* = 0.61; *P* < 0.0001) (Figure 1) and between serum VEGF and IL-6 (*n* = 80; *r* = 0.55; *P* < 0.0001). A correlation was also found between platelet number and serum IL-6 levels (*n* = 76; *r* = 0.36; *P* < 0.0011). There was no correlation between bFGF and any of the other variables.

The comparison of bFGF, VEGF and IL-6 serum levels in patients without thrombocytosis (*n* = 33) versus patients with thrombocytosis (*n* = 13) is given in Table 2. Median VEGF serum levels were 3.2 times higher in the latter group (*P* < 0.0001). Median IL-6 serum levels were 5.8 times higher when thrombocytosis was present (*P* = 0.03). The difference of mean bFGF serum levels was not significant. Comparable results were obtained when median platelet number, i.e. 276 10$^{9}$ l$^{-1}$, was taken as the cut-off value (Table 2).

When the samples were categorized according to the median serum VEGF level, i.e. 314 pg ml$^{-1}$, according to the median platelet number, and according to the detection limit for serum IL-6, the following distributions of samples were found. Of 40 samples with low serum VEGF, 30 also had a low platelet number. Of the 36 samples with high serum VEGF, 26 also had a high platelet number (*n* = 76; $\chi^2$ value = 16.95; *P* < 0.0001). Of the 40 samples with low serum VEGF, 30 also had undetectable low levels of IL-6. Of the 40 samples with high serum VEGF, 33 had serum IL-6 levels above the detection limit (*n* = 80; $\chi^2$ value = 26.60; *P* < 0.0001). Of the 40 samples with a low platelet number, 23 also had undetectable low levels of IL-6. Of the 36 samples with a high platelet number, 23 also had serum IL-6 levels above the detection limit (*n* = 76; $\chi^2$ value = 3.48; *P* = 0.0622).

Platelet number and IL-6 serum levels co-determined VEGF serum levels but not bFGF serum levels (Table 3). Serum VEGF levels were higher (mean serum VEGF of 785 pg ml$^{-1}$) when both platelet number and IL-6 serum levels were high, i.e. above the median value and above the detection limit, respectively, than when only platelet number or only IL-6 was high (mean serum VEGF of 384 pg ml$^{-1}$) (*P* < 0.0001). When serum bFGF was analysed in this way, no differences were observed (Table 3).

*© Cancer Research Campaign 1999*

**Table 4** HUVEC proliferation expressed as the ratio of the number of HUVEC in the wells with the patient serum versus the number of HUVEC in the control wells with fetal calf serum

| HUVEC proliferation (% of control) | bFGF (pg ml$^{-1}$) | VEGF (pg ml$^{-1}$) |
|---|---|---|
| bFGF high and VEGF high (*n* = 8) | | |
| **228** | 10.30 | 763 |
| **200** | 17.00 | 813 |
| **184** | 17.00 | 1490 |
| **160** | 17.00 | 796 |
| **160** | 32.70 | 670 |
| 152 | 11.60 | 767 |
| 152 | 7.51 | 957 |
| 126 | 11.80 | 632 |
| (170 ± 32; 160)a | | |
| bFGF low and/or VEGF low (*n* = 12) | | |
| **155** | 0.49 | 206 |
| 150 | 17.00 | 389 |
| 137 | 0.51 | 105 |
| 136 | 0.55 | 185 |
| 135 | 0.49 | 109 |
| 134 | 1.85 | 172 |
| 134 | 0.87 | 226 |
| 129 | 1.01 | 1130 |
| 126 | 0.31 | 115 |
| 113 | 4.39 | 1650 |
| 97 | 33.00 | 244 |
| 97 | 1.30 | 1160 |
| (128 ± 18; 134)a | | |

The mean proliferation assay result for healthy persons (*n*= 5) was 152% (s.d. 31). Results printed in bold are above this mean value. [a]Between brackets, the mean ± s.d.; median values for HUVEC proliferation (%) are given. Mann–Whitney *U P*-value: 0.0037.

If, as suggested by Banks et al (1998) the platelets are the major source of VEGF in the serum, the theoretical VEGF load of platelets can be calculated by the ratio of VEGF serum concentration and the platelet number. A mean VEGF load of 1.37 pg VEGF $10^{-6}$ platelets was found (s.d. 0.39; range 0.16–3.64; median, 1.16; $n$ = 76). A significant correlation was found between VEGF load and serum IL-6 concentration ($r$ = 0.4; $P$ = 0.0003) (Figure 2). When only samples with a platelet number lower than the median of 276 $10^9$ $l^{-1}$ were analysed, this correlation became stronger ($r$ = 0.67; $P$ < 0.0001). The respective coefficient $r$ for the samples with a high platelet number was 0.39 ($P$ = 0.0171).

When patient sera with a bFGF and a VEGF content higher than the cut-off values we have previously used, i.e. 7.5 pg ml$^{-1}$ and 500 pg ml$^{-1}$ respectively (Dirix et al, 1996; Dirix et al, 1997), were tested, in most cases (five of eight samples) the HUVEC number after 24 h was higher than when the sera of apparently healthy volunteers were used. The opposite was found when patient sera with low bFGF and/or low VEGF content were assayed: only one sample out of 12 resulted in a high HUVEC number in the proliferation assay ($P$ = 0.018). The mean proliferation assay result for the healthy persons was 152%, and was 170% for the patient sera with elevated bFGF and elevated VEGF as compared to 128% for the patient sera with low serum bFGF and/or low serum VEGF content ($P$ = 0.0037) (Table 4).

### DISCUSSION

This study supports the role of platelets in the storage of circulating VEGF, as suggested by Verheul et al (1997), Banks et al (1998) and Wartiovaara et al (1998). A strong correlation was found between platelet count and serum VEGF concentration in a cohort of patients with different types of advanced cancer. The cytokine IL-6 correlated with the total amount of VEGF in the serum and also with the calculated VEGF content of the platelets, indicating a more complex indirect angiogenic action of IL-6 than just the thrombopoietic effect.

As the generation of tumour stroma is remarkably similar to the healing of wounds (Dvorak, 1986), platelet activation, coagulation of extravasated plasma fibrinogen and fibrinolysis are important endpoints of tumour–host cell interactions in growing tumours. Evidence for platelet activation in cancer patients is based on the assessment of circulating levels of β-thromboglobulin, a platelet α-granule component. Levels of β-thromboglobulin were significantly elevated, e.g. in prostatic cancer (Yazaki et al, 1987), in breast cancer (Ferriere et al, 1985) and in small-cell lung cancer (Milroy et al, 1988), compared with those of healthy individuals. VEGF appears to be released by activated platelets together with β-thromboglobulin, suggesting that VEGF is located in the α-granules of platelets (Möhle et al, 1997; Wartiovaara et al, 1998). Clotting of platelet-rich plasma (PRP) prepared from the blood of

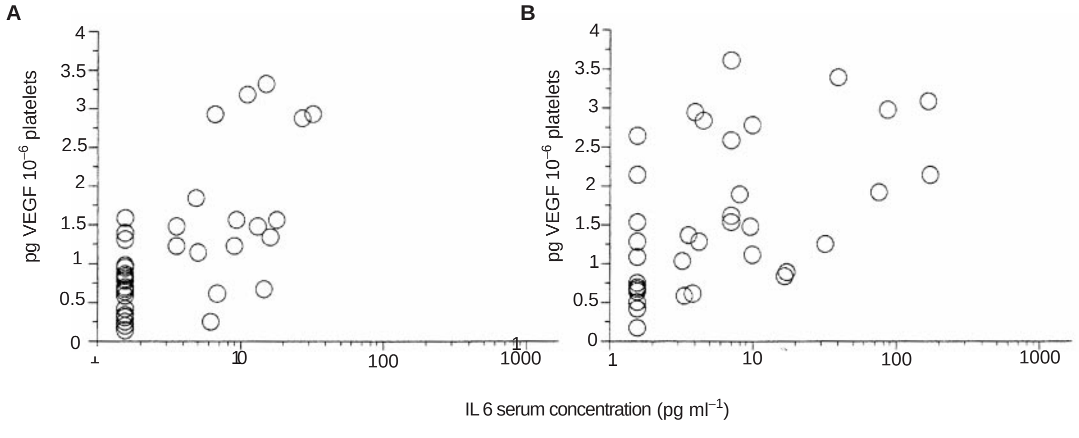

**Figure 2** Theoretical VEGF content of platelets versus IL-6 serum levels for samples with a platelet number lower than the median (A; *n* = 40) and for samples with a platelet number higher than the median (**B**; *n* = 36). Respective *r* and *P*-values are: 0.67/<$10^{-4}$ and 0.39/0.017

*© Cancer Research Campaign 1999*

eight healthy individuals with thrombin resulted in higher VEGF levels than when platelet-free plasma was used (Banks et al, 1998). The VEGF levels obtained after the clotting of the PRP correlated with the respective serum VEGF levels. The theoretical VEGF load of platelets, i.e. the VEGF concentration found after activation of the platelets calculated in terms of the number of platelets present, was highly comparable in PRP and serum. These observations support the hypothesis that platelets are an important source of circulating VEGF. In 27 patients with advanced breast cancer undergoing chemotherapy, a correlation ($r$ = 0.8; $P$ < 0.01) between serum VEGF levels and platelet counts was reported by Verheul et al (1997). In the group of eight healthy individuals (Banks et al, 1998), serum VEGF just failed to achieve a significant correlation with platelet number ($r$ = 0.62; $P$ = 0.1). In our population of 50 patients, corresponding to 80 blood samples, with various types of advanced cancer, a highly significant correlation between platelet number and serum VEGF concentration was found ($r = 0.61$; $P < 10^{-4}$). These results are in accordance with the reduced survival rates of cancer patients with preoperative thrombocytosis, e.g. in lung cancer (Møller Pedersen et al, 1996). We have indeed shown that high serum VEGF, and thus high platelet number, indicates fast growth kinetics in several tumour types (Dirix et al, 1996; Dirix et al, 1997). It is conceivable that platelets provide a means to concentrate the effect of secreted growth factors, e.g. VEGF, onto sites of perturbed integrity of the vascular wall, as present during wound healing and tumour growth. This scavenger function of platelets also conforms to the lack of apparent angiogenesis observed outside of the tumour in patients with high amounts of circulating VEGF protein. Although clear evidence for the endocytic uptake of VEGF by platelets is absent, the incorporation of other circulating proteins into platelet granules and into megakaryocytes has been extensively studied (Handagama et al, 1987; Harrison et al, 1989).

Since plasma VEGF levels are very low (Banks et al, 1998), and probably as a consequence of inefficient inhibition of platelet activation during sample handling, platelets can be regarded as the transporters of the active and stable fraction of circulating VEGF. The in vitro HUVEC proliferation assay results indeed suggest that the concentration of VEGF measured in the serum reflects the biological activity of VEGF. Given the considerable variability of the theoretical VEGF load of platelets (range: 0.16–3.64 pg VEGF $10^{-6}$ platelets), serum VEGF does not seem to be a simple reflection of the number of circulating platelets. As a practical conclusion, we therefore suggest using serum VEGF and not plasma VEGF as suggested by Banks et al (1998), as a measure of circulating VEGF. Since other circulating blood cells may also contain VEGF, measuring VEGF in whole blood might even reflect the active VEGF concentration more accurately (P Salven, personal communication).

We measured the serum IL-6 levels in the cohort of 50 patients with advanced cancer, (1) because IL-6 serum levels correlate with platelet number (Nakano et al, 1998) and intravenous administration of recombinant human IL-6 results in elevation of the platelet number (Clarke et al, 1996); (2) because IL-6 production is increased in tumour tissue (Rak et al, 1994; Degeorges et al, 1996) and during wound healing (Mateo et al, 1994); and (3) because transient tumour-stimulating effects have been observed during IL-6 treatment of cancer patients (Ravoet et al, 1994) and high IL-6 levels are associated with adverse prognosis in several tumour types and benign angiogenic diseases (Blay et al, 1992; Bross et al, 1996).

Serum levels of IL-6 correlate with platelet number and with serum VEGF concentration, but not with serum bFGF concentration. This result can be explained by the thrombopoietic action of IL-6 and the storage of VEGF within platelets. The calculated VEGF content of the platelets is larger when serum IL-6 levels are higher. This result implies an additional effect of IL-6 on VEGF production. Transient expression of IL-6 mRNA was shown in endothelial cells during the physiologic angiogenic processes associated with the development of ovarian follicles and with the embryonic implantation in the maternal decidua (Motro et al, 1990). Treatment of various cell lines, e.g. derived from an epidermoid human carcinoma and a glioma cell line, with IL-6 resulted in the induction of VEGF mRNA (Cohen et al, 1996). The effect of IL-6 on VEGF expression is mediated by specific DNA motifs located on the putative promotor region of VEGF as well as by specific elements in the 5′-untranslated region. Hypoxia has been shown to induce IL-6 mRNA and IL-6 protein in cultured human endothelial cells (Yan et al, 1995). IL-6 can thus be regarded as an indirect angiogenic factor produced by stromal cells and by tumour cells.

Although in benign prostate hyperplasia (Degeorges et al, 1996) and in early-stage radial melanoma growth phase (Rak et al, 1994), stroma-derived IL-6 might function as a growth inhibitor of tumour cells, at least in more advanced cancer, IL-6 might stimulate tumour growth by increasing the amount of active VEGF, stored in the platelets. The stimulation of platelet production by tumour-derived IL-6 favours the endocrine effect of angiogenic factors and facilitates the development of secondary tumours in distant tissues, exemplifying the parasitic nature of cancer.

### REFERENCES

Banks RE, Forbes MA, Kinsey SE, Stanley A, Ingham E, Walters C and Selby PJ (1998) Release of the angiogenic cytokine vascular endothelial growth factor (VEGF) from platelets: significance for VEGF measurements and cancer biology. *Br J Cancer* **77**: 956–964

Blay JY, Negrier S, Combaret V, Attali S, Goillot E, Merrouche Y, Mercatello A, Ravault A, Tourani JM, Moskovtchenko JF and Philip T (1992) Serum levels of interleukin 6 as a prognostic factor in metastatic renal cell carcinoma. *Cancer Res* **52**: 3317–3322

Bousvaros A, Zurakowski D, Fishman SJ, Keough K, Law T, Sun C and Leichter AM (1997) Serum basic fibroblast growth factor in pediatric Crohn's disease. Implications for wound healing. *Dig Dis Sci* **42**: 378–386

Brekken RA, Huang X, King SW and Thorpe PE (1998) Vascular endothelial growth factor as a marker of tumor endothelium. *Cancer Res* **58**: 1952–1959

Bross DA, Leichtner AM, Zurakowski D, Law T and Bousvaros A (1996) Elevation of serum interleukin-6 but not serum-soluble interleukin-2 receptor in children with Crohn's disease. *J Pediatr Gastroenterol Nutr* **23**: 164–171

Clarke D, Johnson PWM, Banks RE, Storr M, Kinsey SE, Johnson R, Morgan G, Gordon MY, lllingworth JM, Perren TJ and Selby PJ (1996) *Cytokine* **8**: 717–723

Cohen T, Nahari D, Weiss Cerem L, Neufeld G and Levi B (1996) Interleukin 6 induces the expression of vascular endothelial growth factor. *J Biol Chem* **271**: 736–741

Degeorges A, Tatoud R, Fauvel-Lafeve F, Podgorniak M-P, Millot G, De Cremoux P and Calvo F (1996) Stromal cells from human benign prostate hyperplasia produce a growth-inhibitory factor for LNCaP prostate cancer cells, identified as interleukin-6. *Int J Cancer* **68**: 207–214

Duensig S, Grosse J and Atzpodien J (1995) Increased serum levels of basic fibroblast growth factor (bFGF) are associated with progressive lung metastases in advanced renal cell carcinoma patients. *Anticancer Res* **15**: 2331–2334

Dvorak HF (1986) Tumors: wounds that do not heal. Similarities between tumor stroma generation and wound healing. *N Engl J Med* **315**: 1650–1659

Dirix LY, Vermeulen PB, Hubens G, Benoy I, Martin M, De Pooter C and Van Oosterom AT (1996) Serum basic fibroblast growth factor and vascular endothelial growth factor and tumour growth kinetics in advanced colorectal cancer. *Ann Oncol* **7**: 843–848

Dirix LY, Vermeulen PB, Pawinski A, Prové A, Benoy I, De Pooter C, Martin M and Van Oosterom AT (1997) Elevated levels of the angiogenic cytokines basic fibroblast growth factor and vascular endothelial growth factor in sera of cancer patients. *Br J Cancer* **76**: 238–243

© Cancer Research Campaign 1999

Dosquet C, Coudert M-C, Lepage E, Cabane J and Richard F (1997) Are angiogenic factors, cytokines, and soluble adhesion molecules prognostic factors in patients with renal cell carcinoma? *Clin Cancer Res* **3**: 2451–2458

Ferriere JP, Bernard D, Legros M, Chassagne J, Chollet P, Gaillard G and Plagne R (1985) β-Thromboglobulin in patients with breast cancer. *Am J Hematol* **19**: 47–53

Folkman J (1990) What is the evidence that tumors are angiogenesis dependent? *J Natl Cancer Inst* **82**: 4–6

Gasparini G (1996) Angiogenesis research up to 1996. A commentary on the state of art and suggestions for future studies. *Eur J Cancer* **32A**: 2379–2385

Handagama PJ, Shuman MA and Bainston DF (1987) Incorporation of intravenously injected albumin, immunoglobulin G, and fibrinogen in guinea pig megakaryocyte granules. *J Clin Invest* **84**: 73–82

Harrison P, Wilbourn B, Debili N, Vainchenker W, Breton-Gorius J, Lawrie AS, Masse J-M, Savidge GF and Cramer EM (1989) Uptake of plasma fibrogen into the alpha granules of human megakaryocytes and platelets. *J Clin Invest* **84**: 1320–1324

Ishibashi T, Kimura H, Shikama Y, Uchida T, Kariyone S, Hirano T, Kishimoto T, Takatsuki F and Akiyama Y (1989) Interleukin-6 is a potent thrombopoietic factor in vivo in mice. *Blood* **74**: 1241–1244

Jaffe EA, Nachman RL, Becker CG, Minick CR (1973) Culture of human endothelial cells derived from umbilical veins. *J Clin Invest* **52**: 2745–2756

Kaushansky K (1995) Thrombopoietin: the primary regulator of platelet production. *Blood* **86**: 419–431

Kumar H, Heer K, Lee PWR, Duthie GS, MacDonald AW, Greenman J, Kerin MJ and Monson JRT (1998) Preoperative serum vascular endothelial growth factor can predict stage in colorectal cancer. *Clin Cancer Res* **4**: 1279–1285

Lake AM, Stauffer JQ and Stuart MJ (1978) Hemostatic alterations in inflammatory bowel disease: response to therapy. *Am J Dig Dis* **23**: 897–902

Liotta LA and Saidel GM (1974) Quantitative relationship of intravascular tumor cells, tumor vessels, and pulmonary metastases following tumor implantation. *Cancer Res* **34**: 997–1004

Mateo RB, Reichner JS and Albina JE (1994) Interleukin-6 activity in wounds. *Am J Physiol* **266**: R1840–1844

Milroy R, Douglas JT, Campbell J, Carter R, Lowe GD and Banham SW (1988) Abnormal haemostasis in small cell lung cancer. *Thorax* **43**: 978–981

Möhle R, Green D, Moore MAS, Nachman RL and Rafii S (1997) Constitutive production and thrombin-induced release of vascular endothelial growth factor by human megakaryocytes and platelets. *Proc Natl Acad Sci USA* **94**: 663–668

Møller Pedersen LM and Milman N (1996) Prognostic significance of thrombocytosis in patients with primary lung cancer. *Eur Respir J* **9**: 1826–1830

Motro B, Itin A, Sachs L and Keshet E (1990) Pattern of interleukin 6 gene expression in vivo suggests a role for this cytokine in angiogenesis. *Proc Natl Acad Sci USA* **87**: 3092–3096

Nakano T, Chaninian AP, Shinjo M, Tonomura A, Miyake M, Togawa N, Ninomiya K and Higashino K (1998) Interleukin 6 and its relationship to clinical parameters in patients with malignant pleural mesothelioma. *Br J Cancer* **77**: 907–912

Rak JW, Hegmann EJ, Lu C and Kerbel RS (1994) Progressive loss of sensitivity to endothelium-derived growth inhibitors expressed by human melanoma cells during disease progression. *J Cell Physiol* **159**: 245–255

Rak J, Filmus J and Kerbel RS (1996) Reciprocal paracrine interactions between tumour cells and endothelial cells: 'angiogenesis progression' hypothesis. *Eur J Cancer* **32A**: 2438–2450

Ravoet C, De Grève J, Vandewoude K, Kerger J, Sculier J-P, Lacor P, Stryckmans P and Piccart M (1994) Tumour stimulating effects of recombinant human interleukin-6. *Lancet* **344**: 1576–1577

Salven P, Teerenhovi L and Joensuu H (1997) A high pretreatment serum vascular endothelial growth factor concentration is associated with poor outcome in non-Hodgkin's lymphoma. *Blood* **90**: 3167–3172

Salven P, Ruotsalainen, Mattson K, and Joensuu H (1998) High pretreatment serum level of vascular endothelial growth factor (VEGF) is associated with poor outcome in small cell lung cancer. *Int J Cancer* **79**: 144–146

Skobe M, Rockwell P, Goldstein N, Vosseler S and Fusenig NE (1997) Halting angiogenesis suppresses carcinoma cell invasion. *Nat Med* **11**: 1222–1227

Vadhan-Raj S, Murray LJ, Bueso-Ramos C, Patel S, Reddy SP, Hoots WK, Johnston T, Papadopoulos NE, Hittelman WN, Johnston DA, Yang TA, Paton VE, Cohen RL, Hellmann SD, Benjamin RS and Broxmeyer HE (1997) Stimulation of megakaryocyte and platelet production by a single dose of recombinant human thrombopoietin in patients with cancer. *Ann Intern Med* **126**: 731–3

Verheul HMW, Hoekman K, Luykx-de Bakker S, Eekman CA, Folman CC, Broxterman HJ and Pinedo HM (1997) Platelet: transporter of vascular endothelial growth factor. *Clin Cancer Res* **3**: 2187–2190

Vermeulen PB, Gasparini G, Fox SB, Toi M, Martin L, McCulloch P, Pezzella F, Viale G, Weidner N, Harris AL and Dirix LY (1996) Quantification of angiogenesis in solid human tumours: an international consensus on the methodology and criteria of evaluation. *Eur J Cancer* **32A**: 2474–2484

Vermeulen PB, Dirix LY, Martin M, Lemmens J and Van Oosterom AT (1997) The predictive value of serum bFGF and VEGF in patients with metastatic renal cell carcinoma treated with interferon α-2b. *J Natl Cancer Inst* **89**: 1317

Wartiovaara U, Salven P, Mikkola H, Lassila R, Kaukonen J, Joukov V, Orpana A, Ristimäki A, Heikinheimo M, Joensuu H, Alitalo K and Palotie A (1998) Peripheral blood platelets express VEGF-C and VEGF which are released during platelet activation. *Thromb Haemost* **80**: 171–175

Weber J, Yang JC, Topalian SL, Parkinson DR, Schwartzentruber DS, Ettinghausen SE, Gunn H, Mixon A, Kim H and Cole D (1993) Phase I trial of subcutaneous interleukin-6 in patients with advanced malignancies. *J Clin Oncol* **11**: 499–506

Yazaki T, Inage H,Iizumi T, Koyama A, Kanoh S, Koiso K, Narita M and Tojo S (1987) Studies on platelet function in patients with prostatic cancer. Preliminary report. *Urology* **30**: 60–63

Yan SF, Tritto I, Pinsky D, Liao H, Huang J Fuller G, Brett J, May L and Stern D (1995) Induction of interleukin 6 (IL-6) by hypoxia in vascular cells. *J Biol Chem* **270**: 11463–11471

© Cancer Research Campaign 1999
